# Efficacy and safety of the human anti-IL-1beta monoclonal antibody canakinumab in rheumatoid arthritis: results of a 12-week, phase II, dose-finding study

**DOI:** 10.1186/1471-2474-12-153

**Published:** 2011-07-07

**Authors:** Rieke Alten, Juan Gomez-Reino, Patrick Durez, Andre Beaulieu, Anthony Sebba, Gerhard Krammer, Ralph Preiss, Udayasankar Arulmani, Albert Widmer, Xavier Gitton, Herbert Kellner

**Affiliations:** 1 Department of Internal Medicine II, Rheumatology, Clinical Immunology, Osteology, Schlosspark-Klinik Teaching Hospital of the Charité, University Medicine Berlin, (Heubnerweg), Berlin, (14059), Germany; 2 Rheumatology and Department of Medicine, Hospital Clinico Universitario, Universidad de Santiago de Compostela, (Travesía da Choupana), Santiago, (15705), Spain; 3 Rheumatology Department, Cliniques Universitaires Saint-Luc, Université Catholique de Louvain, (Av. Hippocrate), Brussels, (1200), Belgium; 4 Centre de Rheumatologie St-Louis, (100-1200 Germain-des-Prés Ave.). Quebec, (QC G1V 3M7), Canada; 5 Arthritis Associates, (36338 Us Highway), Palm Harbor, (34684) Florida, USA; 6 Clinical Development, Novartis Pharma AG, (Lichtstr.), Basel, (4056), Switzerland; 7 Clinical Development, Novartis Pharmaceuticals, (Health Plaza), East Hanover, (07936), New Jersey, USA; 8 Facharzt für Innere Medizin Rheumatologie and Gastroenterologie, Schwerpunktpraxis für Rheumatologie und Gastroenterologie, (Romanstr. 9), Muenchen, (80639) Germany

## Abstract

**Background:**

Canakinumab is a fully human anti-interleukin IL-1beta monoclonal antibody, being investigated for the treatment of rheumatoid arthritis (RA). This multicenter, phase II, randomized, double-blind, placebo-controlled, parallel-group, dose-finding study investigated the efficacy and safety of canakinumab in patients with active RA despite ongoing therapy at stable doses of methotrexate.

**Methods:**

Patients were randomized to receive one of four regimens, in addition to methotrexate, for 12 weeks: canakinumab 150 mg subcutaneously (SC) every 4 weeks (q4wk), canakinumab 300 mg SC (2 injections of 150 mg SC) every 2 weeks, a 600 mg intravenous loading dose of canakinumab followed by 300 mg SC every 2 weeks', or placebo SC every 2 weeks.

**Results:**

Among 274 patients with evaluable efficacy data, the percentage of responders according to American College of Rheumatology 50 criteria (the primary endpoint, based on a 28-joint count) was significantly higher with canakinumab 150 mg SC q4wk than with placebo (26.5% vs. 11.4%, respectively; p = 0.028). Compared to placebo, this dosage of canakinumab was also associated with significantly more favorable responses at week 12 with respect to secondary endpoints including the Disease Activity Score 28, scores on the Health Assessment Questionnaire and Functional Assessment of Chronic Illness Therapy-Fatigue, swollen 28-joint count, and patient's and physician's global assessments of disease activity. No safety concerns were raised with canakinumab therapy, particularly with regard to infections. Few injection-site reactions occurred.

**Conclusion:**

The addition of canakinumab 150 mg SC q4wk improves therapeutic responses among patients who have active RA despite stable treatment with methotrexate.

**Trial Registration:**

(ClinicalTrials.gov identifier: NCT00784628)

## Background

Rheumatoid arthritis (RA) is a chronic autoimmune disease that can lead to progressive joint destruction and disability [1]. In the past decade, the use of disease-modifying antirheumatic drugs (DMARDs), including methotrexate, has been recognized as the most effective therapy for RA [1]. Low-dose weekly methotrexate substantially improves remission rates and has become the most widely prescribed DMARD [1].

The development of biologic DMARDs has ushered in a new therapeutic era based on improved knowledge of the pivotal roles of pro-inflammatory cytokines (*e.g*., tumor necrosis factor [TNF]-α and several interleukins [ILs], such as IL-6 and IL-1beta) in RA [2,3]. In several animal models, the administration of antibodies against IL-1 has been shown to protect against systemic and local inflammation (*e.g*., arthritis) and to decrease the histopathological findings of inflammation and osteoarticular destruction [2]. IL-1beta is involved in inflamed synovial tissue from RA patients, and increased levels of IL-1beta have been documented in the synovial fluid of patients with RA [3,4]. Treatment with recombinant IL-1 receptor antagonist (IL-1Ra) anakinra has been shown to be effective in RA; however, its efficacy seems to be lower as compared to TNF-α inhibitors [5], and its administration is frequently associated with injection-related adverse events (AEs) [5].

Canakinumab is a fully human anti-IL-1beta monoclonal antibody with a plasma half-life of 3-4 weeks that selectively neutralizes the bioactivity of IL-1beta. This agent has recently been approved by the US Food and Drug Administration (FDA), by the European Medicines Agency, in Switzerland, and in other countries for the treatment of another IL-1beta-driven disease, cryopyrin-associated periodic syndrome, in which it has demonstrated significant and long-lasting efficacy [6]. Further studies have been published showing efficacy of canakinumab in systemic juvenile idiopathic arthritis (SJIA)[7] and in gouty arthritis in treating pain, signs, and symptoms of inflammation and preventing recurrent flares [8,9].

Canakinumab was previously assessed as an add-on therapy in a randomized, double-blind, placebo-controlled, dose-escalation, proof-of-concept study involving 53 patients with active RA despite ongoing treatment with a stable dose of methotrexate (≥ 15 mg/wk for ≥ 3 months) [10]. Analyses of responses to intravenous (IV) doses of 0.3, 1.3, and 10 mg/kg revealed that the highest dose of canakinumab significantly reduced disease activity (six patients reached American College of Rheumatology [ACR] 20, three ACR 50 and two ACR 70) by day 43. Other findings included onset of action within 3 weeks, normalization in C-reactive protein (CRP) levels, and a good tolerability profile including very few to no injection-site reactions.

In light of these observations, a trial was undertaken to assess the efficacy and safety of three canakinumab dose regimens as add-on therapy in patients with active RA despite the use of maximum tolerated doses of methotrexate.

## Methods

### Study design

This trial was designed as a phase II, 12-week, randomized, double-blind, placebo-controlled, parallel-group, dose-finding study of the efficacy and safety of additional canakinumab in patients receiving methotrexate for RA. The study was conducted at 56 centers in Europe and North America from November 2006 to September 2008. The primary objective of this trial was to assess the efficacy of three dose regimens of canakinumab compared to placebo as add-on treatment in patients who had active RA despite stable treatment with methotrexate at the maximum tolerated dose (≤ 25 mg/week). Secondary objectives were to evaluate the onset of effect of canakinumab; its effect on components of the ACR criteria, including a marker of inflammation (high-sensitivity CRP [hsCRP]) vs. placebo after 12 weeks; its immunogenicity after 12 weeks of repeated exposure; its pharmacokinetics and pharmacodynamics (to contribute to decision-making for phase III studies); and its overall safety and tolerability.

### Patients

Patients were considered eligible for participation in the trial if they were males or females ≥ 18 years of age who met the revised 1987 ACR classification criteria for RA and had symptoms for ≥ 3 months before randomization [11]. Active RA was defined as ≥ 6 of 28 tender and swollen joints with hsCRP ≥ 10 mg/L and/or erythrocyte sedimentation rate (ESR) ≥ 28 mm over the first hour, at the time of randomization. Patients were also required to be in functional status classes I, II, or III according to the ACR 1991 revised criteria [12]. Participants were required to have been treated with methotrexate at the maximum tolerated dose (≤ 25 mg/week) and at a stable dose of ≥ 7.5 mg/week for ≥ 12 weeks before randomization. Patients who had failed treatment with any DMARD, including any such agent used in combination with methotrexate as well as any biologic agent, were eligible for participation after an appropriate washout period before enrollment. Patients taking systemic corticosteroids, nonsteroidal anti-inflammatory drugs (NSAIDs), including cyclooxygenase-2 inhibitors, or paracetamol/acetaminophen had to have been on stable doses for at least 4 weeks before randomization. The maximum allowable dose of systemic corticosteroids was ≤ 10 mg/day prednisone or an equivalent for ≥ 4 weeks.

Subjects were excluded from participation if they had previously experienced hypersensitivity to the study drug or to molecules with similar structures, had undergone intra-articular therapy for RA within the previous 4 weeks, were pregnant or breastfeeding, or had a positive purified protein derivative of tuberculin skin test without a follow-up negative chest X-ray.

### Treatment

Following a screening period of 3 days to 4 weeks, patients were randomized in a double-blind fashion to receive one of the following four possible treatments in addition to methotrexate over 12 weeks: canakinumab 150 mg subcutaneously (SC) every 4 weeks (q4wk), canakinumab 300 mg SC (2 injections of 150 mg SC) every 2 weeks (q2wk), a 600 mg IV loading dose of canakinumab followed by 300 mg SC q2wk, or placebo SC q2wk. The canakinumab doses were selected based on the results of a previous dose-escalation, proof-of-concept study in patients with active RA despite maximum tolerated doses of methotrexate, as well as on pharmacokinetic/pharmacodynamic modeling [10].

### Study endpoints

The primary efficacy endpoint was the response to treatment according to ACR 50 criteria at 12 weeks. Calculations were based on 28-joint counts [13]. Secondary efficacy variables included responses according to the ACR 20 and ACR 70 at week 12; ACR 20, ACR 50, and ACR 70 responses at any visit; ACR component variables; Short Form-36 (SF-36^®^); Functional Assessment of Chronic Illness Therapy-Fatigue (FACIT-F^©^); Disease Activity Score 28 (DAS28, as well as DAS-based European League Against Rheumatism [EULAR] criteria); and Health Assessment Questionnaire (HAQ^©^).

Samples were taken during the study visits (no pre-defined time of the day was requested for this) from a forearm vein (via direct venipuncture or from an indwelling cannula) into a sodium or lithium heparin tube at each time point. Soluble plasma protein markers related to the targeted pathway were measured with a multiplex panel (including among others IL-6, vascular endothelial growth factor, or IL-1Ra).

Safety was assessed on the basis of AEs and serious adverse events (SAEs); occurrence of infection; hematologic parameters; blood biochemistry; urinalysis; evaluations of antinuclear antibody, and anti-double-stranded DNA antibody; vital signs; and tolerability. Spleen sonography was included in the protocol in response to a request from the FDA, which had expressed concern regarding the fact that spleen weight was higher in treated male marmosets than in controls during a 13-week SC experimental study, even though this occurrence was not replicated in a subsequent 26-week IV toxicology study [Unpublished data on file at Novartis Pharma AG, Basel, Switzerland]. Pharmacokinetic/pharmacodynamic parameters and immunogenicity were also examined and will be reported elsewhere.

### Statistical analyses

Descriptive statistics were used to summarize demographics and safety by treatment regimen and, where appropriate, by time point. The primary efficacy variable, ACR 50 responder rate, was tested for superiority in canakinumab treatment groups vs. placebo group in the intent-to-treat population. Each of the canakinumab groups was compared against the placebo group based on a logistic regression model with treatment, center, and baseline DAS28-CRP as covariates. Missing values were imputed using the last-observation-carried-forward approach. Secondary variables (DAS28, CRP, and all ACR component variables) were analyzed using an analysis of covariance fixed-effects model with treatment as main effect and correcting for the covariates center and baseline value.

Calculation of sample size determined that 63 patients would need to be randomized to each treatment group in order to have 90% power to show a significant difference between canakinumab treatment groups and placebo at a 5% level of significance in a 2-sided Fisher's exact test. The data were analyzed using version 8.2 of the SAS statistical package.

### Ethical conduct

The study and any amendments were reviewed by the independent ethics committee or institutional review board for each center. The study was conducted according to the ethical principles of the Declaration of Helsinki. Written informed consent for the study procedures was obtained from each patient before initiating any study-specific procedure. (ClinicalTrials.gov identifier: NCT00784628)

## Results

### Patients

**Figure 1:**
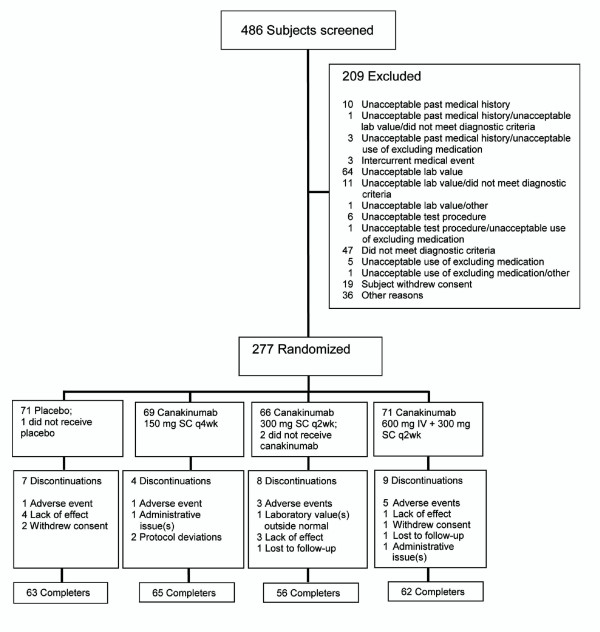
**Study flow**.

Of the 486 patients screened, 209 were excluded in accordance with enrollment criteria and 277 were randomized to one of the four treatment groups (Figure 1). A total of 274 patients were treated and had post-baseline efficacy data recorded (three patients were randomized but not treated due to study discontinuation on the day of randomization).

Demographic and baseline characteristics of the study participants are summarized in Table 1. Patients randomized to canakinumab 300 mg SC q2wk had the highest mean age (61 years). The treatment groups were comparable with respect to duration of RA, number of DMARDs previously received, and functional status. The groups had comparable baseline values with regard to disease characteristics evaluated by DAS28, FACIT-F, and HAQ, as well as rheumatoid factor (RF), SF-36, ESR, hsCRP, and 100-mm visual analog scale (VAS) for pain and for physician's and patient's global assessments of disease activity.

**Table 1:** Baseline demographic and disease characteristics

Variable	Canakinumab 150 mg SC q4wk, n = 69	Canakinumab 300 mg SC q2wk, n = 64	Canakinumab 300 mg SC q2wk + 600 mg IV loading dose, n = 71	Placebo, n = 70
Female sex, n (%)	56 (81.2)	57 (89.1)	60 (84.5)	52 (74.3)

Age, mean (SD) years	57.10 (11.899)	61.02 (12.244)	55.62 (11.236)	57.53 (12.121)

Duration of RA, mean (SD) years	11.10 (9.476)	10.13 (8.413)	9.83 (8.144)	8.77 (8.837)

Prior DMARDs, n (%)*				

0	51 (73.9)	52 (81.3)	50 (70.4)	54 (77.1)

1	15 (21.7)	11 (17.2)	15 (21.1)	13 (18.6)

> 1	3 (4.4)	1 (1.6)	6 (8.7)	3 (4.3)

DAS28, mean (SD) score	5.937 (0.7644)	5.818 (0.837)	5.978 (0.7151)	5.870 (0.8002)

RF (kIU/L), mean (SD)	250.9 (920.49)	259.4 (396.15)	154.6 (229.11)	180.6 (311.45)

HAQ, mean (SD) score	1.643 (0.6106)	1.572 (0.6107)	1.613 (0.4879)	1.566 (0.5565)

Number of tender joints, mean (SD)	15.6 ± 6.00	15.2 ± 6.00	16.8 ± 6.20	16 ± 5.87

Number of swollen joints, mean (SD)	11.8 (4.29)	11.9 (4.39)	12.4 (4.95)	12.1(4.75)

Abbreviations: DAS28, Disease Activity Score 28, DMARD, disease-modifying antirheumatic drugs; HAQ, Health Assessment Questionnaire; IV, intravenous; q2wk, every 2 weeks; q4wk, every 4 weeks; SD, standard deviation; RA, rheumatoid arthritis; RF, rheumatoid factor

### Efficacy

**Figure 2:**
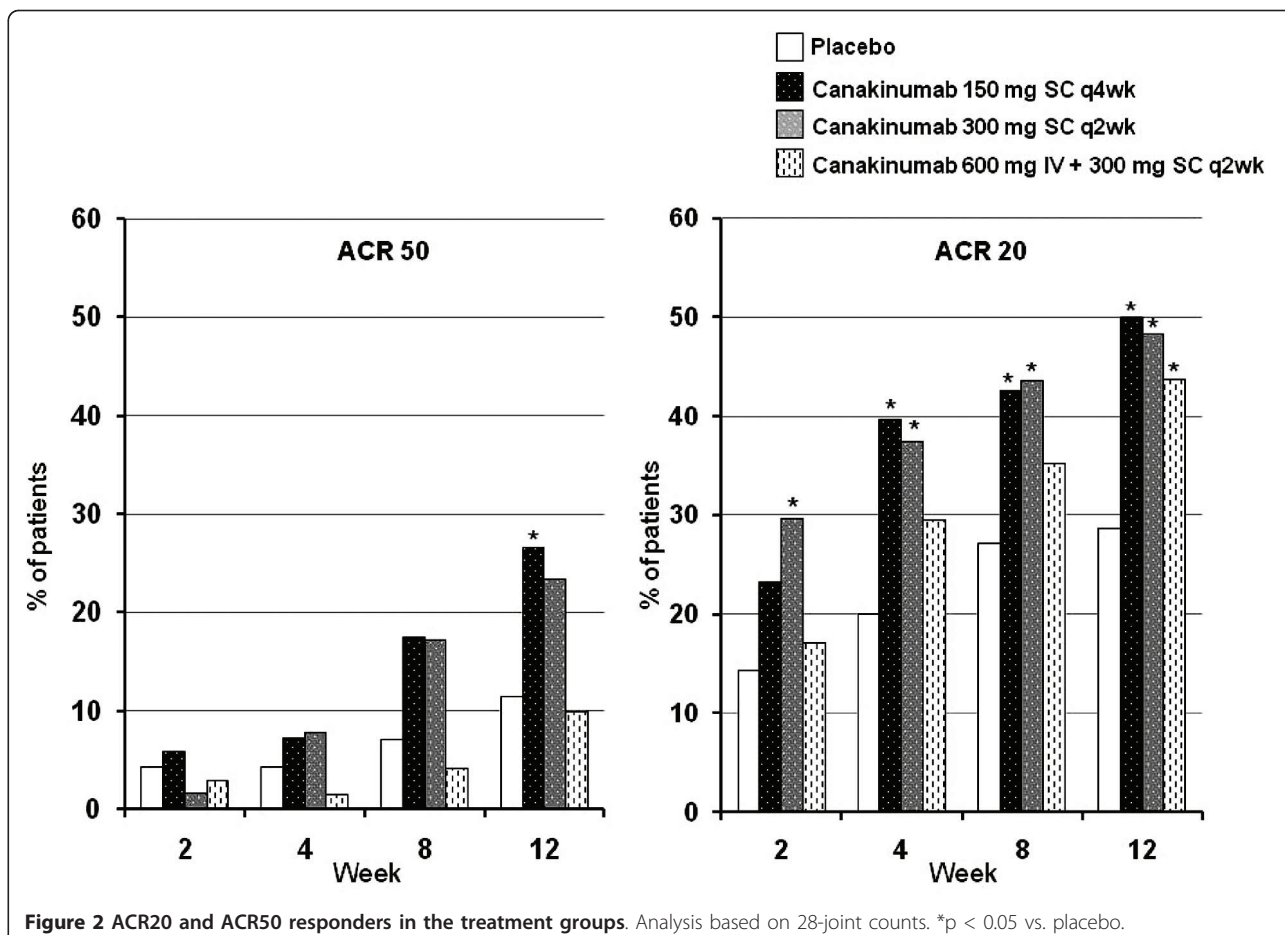
**ACR20 and ACR50 responders in the treatment groups**. Analysis based on 28-joint counts. *p < 0.05 vs. placebo.

As shown in Figure 2, the percentage of ACR 50 responders at week 12 (the primary endpoint) was significantly higher with canakinumab 150 mg SC q4wk than placebo (26.5% vs. 11.4%, respectively; p = 0.028). The response rate was also higher in patients receiving canakinumab 300 mg SC q2wk (23.4%), but the difference vs. placebo was not significant. No statistically significant differences were found for the numbers of ACR 50 responders at week 12 in the 600 mg IV plus 300 mg SC q2wk group or the 300 mg SC q2wk group compared to the placebo group.

Percentages of ACR 20 responders in the canakinumab and placebo groups (a secondary endpoint) are shown in Figure 2. Over the course of the study, percentages of ACR 70 responders were higher in the canakinumab 150 mg SC q4wk group than in the placebo group except at week 2. Percentages of ACR 70 responders in the canakinumab 300 mg SC q2wk group were higher than in the placebo group at all visits. No significant differences between any of the canakinumab dosage groups and the placebo group were observed with regard to ACR 70 response rates at 12 weeks.

**Figure 3:**
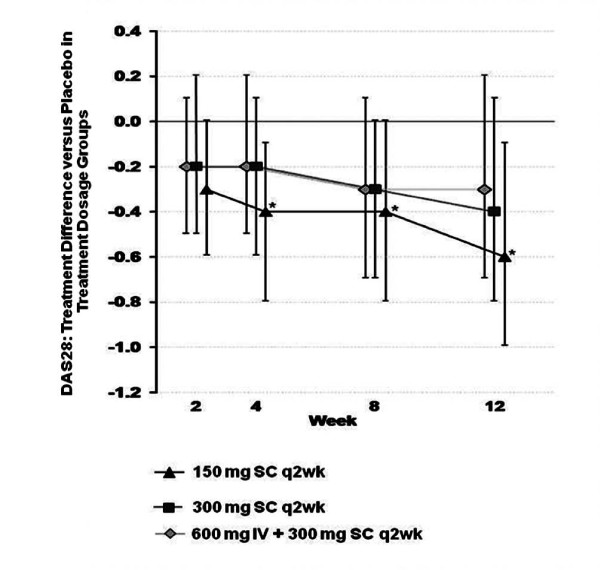
**DAS28: Treatment difference between canakinumab groups and placebo (using last observation carried forward), by visit (intention-to-treat population)**. *p < 0.04 vs. placebo

Significant differences favoring canakinumab 150 mg SC q4wk vs. placebo were apparent with respect to the swollen 28-joint count and patient's and physician's global assessments of disease activity (p < 0.05 for all comparisons; Table 2), as well as the HAQ and DAS28 scores (p < 0.05 for both comparisons; Table 3 and Figure 3, respectively). The percentage of patients with a good response on the DAS-based EULAR criteria was likewise higher with canakinumab 150 mg SC q4wk (25.0%) than with 300 mg SC q2wk (18.8%), 600 mg IV loading dose plus 300 mg SC q2wk (12.7%), or placebo (18.6%). Across the weeks of the study, greater average decreases in ESR levels compared to baseline were observed in the three canakinumab treatment groups than in the placebo group.

**Table 2:** Response criteria: LS mean between-treatment difference for canakinumab vs placebo at week 12

Variable	Canakinumab 150 mg SC q4wk, n = 69	Canakinumab 300 mg SC q2wk, n = 64	Canakinumab 300 mg SC q2wk + 600 mg IV loading dose, n = 71
Number of tender joints			

Baseline, mean (SD)	15.6 (6.00)	15.2 (6.00)	16.8 (6.20)

Difference vs. placebo in LS mean change (95% CI)	-1.7 (-4.1, 0.6)	-0.3 (-2.7, 2.2)	0.2 (-2.1, 2.6)

p-value	0.152	0.837	0.839

Number of swollen joints			

Baseline, mean (SD)	11.8 (4.29)	11.9 (4.39)	12.4 (4.95)

Difference vs. placebo in LS mean change (95% CI)	-1.8 (-3.6, -0.1)	-1.9 (-3.7, -0.1)	-1.2 (-3.0, 0.6)

p-value	0.040*	0.039*	0.178

RA pain intensity, VAS in mm			

Baseline, mean (SD)	61.6 (18.00)	59.7 (19.82)	65.0 (16.21)

Difference vs. placebo in LS mean change (95% CI)	-6.5 (-14.0, 1.1)	-4.7 (-12.3, 3.0)	0.2 (-2.1, 2.6)

p-value	0.091	0.233	0.336

Physicians' global assessment of disease activity, VAS in mm			

Baseline, mean (SD)	65.4 (15.14)	60.1 (17.46)	64.7 (12.93)

Difference vs. placebo in LS mean change (95% CI)	-8.8 (-16.6, -0.9)	-6.4 (-14.4, 1.7)	-4.0 (-11.8, 3.8)

p-value	0.029*	0.121	0.316

Patients' global assessment of disease activity, VAS in mm			

Baseline, mean (SD)	63.4 (16.97)	63.6 (20.67)	67.8 (15.94)

Difference vs. placebo in LS mean change (95% CI)	-7.5 (-14.7, -0.3)	-7.4(-14.7, 0.0)	-4.5 (-11.7, 2.8)

p-value	0.041*	0.049*	0.226

hsCRP, mg/L			

Baseline, mean (SD)	16.62 (18.735)	16.60 (17.050)	6.59 (20.785)

Difference vs. placebo in LS mean change (95% CI)	-7.3 (-12.5, -2.1)	-2.7 (-8.1, 2.6)	-4.9 (-10.1, 0.3)

p-value	0.007*	0.313	0.065

*Statistical significance (2-sided) vs. placebo. Statistical model is analysis of covariance adjusting for treatment and center with baseline value as a covariate

Abbreviations: CI, confidence interval; hsCRP, high-sensitivity C-reactive protein; IV, intravenous; LS mean, least-squares mean; q2wk, every 2 weeks; q4wk, every 4 weeks; SC, subcutaneous; SD, standard deviation; VAS, visual analog scale

**Table 3:** Response criteria: LSM difference between canakinumab and placebo for HAQ and FACIT-F scores at week 12

Variable	Canakinumab 150 mg SC q4wk, n = 69	Canakinumab 300 mg SC q2wk, n = 64	Canakinumab 300 mg SC q2wk + 600 mg IV loading dose, n = 71
HAQ score			

Baseline, mean (SD)	1.643 (0.6106)	1.572 (0.6106)	1.613 (0.4879)

Difference vs. placebo in LS mean change (95% CI)	-0.2 (-0.3, 0.0)	-0.1 (-0.3, 0.1)	-0.1 (-0.3, 0.0)

p-value	0.036*	0.253	0.171

FACIT-F score			

Baseline, mean (SD)	25.6 (12.51)	25.8 (11.87)	22.5 (9.26)

Difference vs. placebo in LS mean change (95% CI)	4.4 (1.3, 7.5)	2.5 (-0.7, 5.6)	3.5 (0.4, 6.7)

p-value	0.006*	0.120	0.028*

*Statistical significance (2-sided) vs. placebo. Statistical model is analysis of covariance adjusting for treatment and center with baseline value as a covariate

Abbreviations: CI, confidence interval; FACIT-F, Functional Assessment of Chronic Illness Therapy--Fatigue; HAQ, Health Assessment Questionnaire; IV, intravenous; LSM, least-squares mean; q2wk, every 2 weeks; q4wk, every 4 weeks; SC, subcutaneous; SD, standard deviation

Compared to the placebo group, all three canakinumab treatment groups exhibited trends toward greater improvement in FACIT-F and SF-36 scores (Tables 3 and 4, respectively). The most favorable changes were apparent among patients receiving the 150 mg SC q4wk regimen. At week 12, notable improvement in all canakinumab treatment groups compared to the placebo group was observed for the SF-36 subscales for bodily pain, physical functioning, vitality, social functioning, and mental health. Compared to placebo, significantly greater improvement in FACIT-F scores was observed with canakinumab 150 mg SC q4wk, and the 600 mg IV loading dose plus 300 mg SC q2wk.

**Table 4:** Response criteria: SF-36 scores at baseline and at study assessment time points

Variable	Canakinumab 150 mg SC q4wk, n = 71	Canakinumab 300 mg SC q2wk, n = 64	Canakinumab 300 mg SC q2wk + 600 mg IV loading dose, n = 69	Placebo, n = 70
Mean SF-36 physical component summary score				

Baseline, mean (SD)	27.19 (7.092)	28.57 (7.497)	26.29 (6.080)	27.48 (6.616)

Week 2, mean (SD)	29.70 (7.944)	28.94 (7.503)	27.86 (6.441)	29.58 (8.438)

Week 4, mean (SD)	30.40 (8.400)	30.87 (8.608)	28.91 (8.445)	29.94 (8.413)

Week 8, mean (SD)	31.48 (9.833)	31.32 (8.230)	28.89 (7.322)	29.44 (8.569)

Week 12, mean (SD)	32.92 (9.922)	31.62 (8.752)	30.33 (7.129)	30.17 (9.221)

Mean SF-36 mental component summary score				

Baseline, mean (SD)	44.38 (12.891)	43.40 (11.325)	41.61(10.845)	44.64(12.779)

Week 2, mean (SD)	45.94 (12.518)	46.02 (11.083)	42.37 (10.585)	45.09 (12.834)

Week 4, mean (SD)	46.61 (12.469)	46.14 (12.008)	43.85 (11.433)	46.16 (12.835)

Week 8, mean (SD)	47.41 (12.228)	46.22 (12.762)	44.09 (11.849)	45.82 (12.032)

Week 12, mean (SD)	48.44 (12.421)	46.74 (11.595)	43.05 (11.958)	44.99 (12.294)

Abbreviations: IV, intravenous; q2wk, every 2 weeks; q4wk, every 4 weeks; SC, subcutaneous; SF-36, Short Form-36; SD, standard deviation

### Biomarkers

The measurement of soluble plasma protein markers related to the targeted pathway did not elucidate any biomarker predictors of response. However, blood sampling for biomarkers was not undertaken at a consistent time.

### Safety

A total of 246 patients completed the study. Reasons for discontinuation are shown in Figure 1. The proportion of patients who experienced at least one AE was 52.6% in the overall study population and was lower with canakinumab 150 mg SC q4wk than in the other three treatment groups (Table 5). AEs were mostly mild to moderate in intensity and did not appear to be related to either dose or age. SAEs or clinically significant AEs were reported in 4.7% of the overall study population, with a lower incidence in the canakinumab 150 mg SC q4wk group than in the other three groups. No deaths occurred. The number of discontinuations due to AEs was likewise lowest with canakinumab 150 mg SC q4wk.

**Table 5:** Adverse events in the safety population

Parameter	Canakinumab 150 mg SC q4wk, n = 69	Canakinumab 300 mg SC q4wk, n = 64	Canakinumab 300 mg SC q2wk + 600 mg IV loading dose, n = 71	Placebo, n = 70
Patients with at least 1 AE, n (%)	32 (46.4)	35 (54.7)	40 (56.3)	37 (52.9)

Patients with serious AEs, n (%)*	1 (1.4)	4 (6.3)	3 (4.2)	5 (7.1)

Discontinuations due to AEs, n (%)	1 (1.4)	3 (4.7)	5 (7.0)	2 (2.9)

Most frequent AEs (in ≥ 5% of patients) Bronchitis, n (%)	-	4 (6.3)	-	-

Nasopharyngitis, n (%)	4 (5.8)	4 (6.3)	4 (5.6)	4 (5.7)

Upper respiratory tract infection, n (%)	-	-	4 (5.6)	-

*Serious AEs: canakinumab 150 mg SC q4wk--thoracic vertebral fracture (n = 1); canakinumab 300 mg SC q2wk--cellulitis, abscess limb (n = 1), diverticulitis (n = 1), soft tissue infection (n = 1); canakinumab 600 mg IV plus 300 mg SC q2wk, gastritis (n = 1), urosepsis (n = 1); placebo--abdominal hernia, obstructive (n = 1), atrial fibrillation (n = 1), gastric ulcer hemorrhage (n = 1), RA flare (n = 2)

Abbreviations: AE, adverse event; IV, intravenous; q2wk, every 2 weeks; q4wk, every 4 weeks; RA, rheumatoid arthritis; SC, subcutaneous

No clinically meaningful changes were observed in the hematologic parameters or in biochemistry or urinary measurements, relative to baseline. Three patients (4.2%) in the group receiving canakinumab 600 mg IV plus 300 mg SC had elevations in alanine transaminase (ALT) or aspartate transaminase (AST) ≥ 3 times the upper limit of normal (ULN). Among these three patients, only one had an ALT elevation ≥ 3 times the ULN, and two had elevations in both ALT and AST ≥ 3 times the ULN. In the group receiving canakinumab 300 mg SC q2wk, two patients had ALT/AST elevations: one patient (1.6%) had an ALT ≥ 5 times the ULN and AST ≥ 3 times the ULN, another patient (1.6%) had an ALT ≥ 5 times ULN. The patient with ALT ≥ 5 times discontinued the study due to the ALT elevation, for this patient, an elevated ALT of 1.5 times ULN at baseline was noted. In all patients, except the patient who discontinued, liver enzyme elevations were transient and returned to normal values during the study. No patients had ALT/AST elevations with accompanying elevation of total bilirubin. Spleen sonography showed no significant differences in spleen size between the canakinumab and placebo groups. Most patients had no injection-site reactions. An absence of a tolerability reaction at any time during the trial was recorded for 92.8% of the 150 mg SC q4wk group, 93.8% of the 300 mg SC q2wk group, 94.4% of the 300 mg SC q2wk plus 600 mg IV loading dose group, and 95.7% of the placebo group. Mild or moderate injection-site reactions were reported in 7.2% of the 150 mg SC q4wk group, 6.3% of the 300 mg SC q2wk group, 5.6% of the 300 mg SC q2wk plus 600 mg IV loading dose group, and 2.9% of the placebo group. No severe reactions occurred.

## Discussion

The results of this study demonstrated the efficacy of additional treatment with canakinumab (150 mg SC q4wk vs. placebo) in patients with active RA despite stable treatment with methotrexate. The primary endpoint of this study ACR 50 improvement compared to placebo was reached with the 150 mg sc q4wk dose. Efficacy was also confirmed by the analyses of secondary endpoints (*i.e.*, CRP, HAQ, and DAS 28). The response to this dose of canakinumab increased over time, suggesting that further improvement may be seen with longer-term treatment. The canakinumab regimen of 150 mg SC q4wk was also well tolerated, and the safety profile was comparable to that of placebo. Fewer than 8% of the patients had injection-site reactions, which were mild and of short duration. No unusual or opportunistic infections were observed with canakinumab in comparison to placebo during this short-term period study.

No dose effect was seen in this study and the canakinumab group receiving the additional loading dose did not demonstrate an increase in efficacy. It is possible that therapy led to differential up-or down-regulation of receptors or soluble receptors, including type 2 decoy receptors, involved in regulation of IL-1beta activity, thereby resulting in paradoxical effects. Another factor could have been the relatively modest sample size. The results could be consistent with a subgroup of canakinumab-responsive patients, although randomization should have ensured equal distribution amongst dosing groups. It would be of great importance to identify biomarkers for patients with a good response to canakinumab, although our biomarker panel failed to identify any, potentially limited by technical factors. For example, diurnal variation in biomarker levels, such as IL-6, may have influenced the outcome.

The biological role of IL-1β in the disease pathogenesis of RA is not fully understood. Data from anakinra studies suggest that there might be a relatively inferior biological role of IL-1beta as compared to TNF-α in this disease. In a meta-analysis by the Cochrane group including 5 randomized trials involving 2876 patients (781 on placebo, 2065 on anakinra), anakinra 50-100 mg per day improved symptoms of pain, function, and stiffness over a 6-month period [5]. Significant improvements were noted for ACR 20 (38% vs 23% on placebo), which were considered clinically meaningful, although modest. ACR 50 was achieved by 18% vs 7%, and ACR 70 by 7% vs 2% of patients. The ACR 50 rates achieved in our study within 12 weeks in the canakinumab 150 mg q4wk group (26% vs 11% canakinumab vs placebo) compare favorably with the outcomes reported for anakinra.

In contrast, in SJIA IL-1β plays a major role in disease pathology in the majority of patients [7]. In addition, IL-1beta plays also a key role in gouty arthritis inflammation, making targeted anti-IL-1beta therapy an appropriate option [14,15]. In a phase II dose-ranging study in patients with acute gouty arthritis who were unable to receive NSAIDs and/or colchicine, canakinumab provided more rapid and sustained pain relief and significantly reduced the risk of new flares compared with triamcinolone acetonide 40 mg [8].

The findings from this study show that canakinumab is able to significantly increase the proportion of RA patients with ACR 50 responses at 12 weeks, even though the magnitude of effect in the overall study population was relatively small. The study was subject to certain limitations, such as rather small sample sizes per treatment group and short duration of treatment. Some patients still do not respond to current biologics, reflecting an unmet medical need. The identification of patients who will respond remains a challenge. The fact that different patients experienced a highly favorable response to canakinumab could lead to the detection of predictive biomarkers of response in these patients in future studies.

## Conclusion

Canakinumab 150 mg sc q4wk demonstrated significant efficacy in a subset of RA patients refractory to methotrexate, even though the effect size in the overall study population was small. There were no safety concerns (and, notably, no sign of an increased infection rate). No predictor-of-response biomarker could be discerned in responders. The identification of biomarkers in responding patients with RA will hopefully be elucidated by future research and will allow the next step in the direction of personalized medicine and elucidate the role of the anti-IL-1β antibody canakinumab.

## Competing interests

Dr. Rieke Alten has received honoraria for speaking from Novartis. Dr. Anthony Sebba is a member of the speakers' bureau and has received speaker fees from Novartis. Gerhard Krammer, Dr. Ralph Preiss, Dr. Udayasankar Arulmani, Albert Widmer and Dr. Xavier Gitton are employees of Novartis and own shares in the company. All other authors have no conflict of interest.

## Authors' contributions

RA, PD, AB, GK, AW, XG contributed to the clinical trial design; JGR, AS, and HK were involved in the data collection; RA, AS, and HK were investigators; JGR, XG, GK, AW, RP, and UA were involved in the data analysis and data interpretation. All authors have participated in writing and reviewing and have approved the paper for publication.

## Authors' information

Dr. Rieke Alten, Chief of the internal medicine division at Schlosspark Clinic in Berlin

Dr. Juan Gomez-Reino, Professor of Medicine, Head of Rheumatology Service

Dr. Patrick Durez, Chef de Clinique

Dr. Andre Beaulieu, Rheumatologist

Dr. Anthony Sebba, Assistant Clinical Professor

Gerhard Krammer, M.Sc., Program Section Leader

Dr. Ralph Preiss, Global Medical Director

Dr. Udayasankar Arulmani, Senior Medical Scientific Expert

Albert Widmer, M.Sc, Expert Statistician

Dr. Xavier Gitton, Senior Global Team Director

Dr. Herbert Kellner, Professor of Rheumatology

## Pre-publication history

The pre-publication history for this paper can be accessed here:

http://www.biomedcentral.com/1471-2474/12/153/prepub
